# Longitudinal analysis of primary and secondary factors related to fatigue in multiple sclerosis

**DOI:** 10.1007/s13760-020-01545-6

**Published:** 2020-11-13

**Authors:** Jana Schließeit, Frederike Cosima Oertel, Graham Cooper, Alexander U. Brandt, Judith Bellmann-Strobl

**Affiliations:** 1 Experimental and Clinical Research Center, Max-Delbrück-Centrum für Molekulare Medizin and Charité—Universitätsmedizin Berlin, Corporate Member of Freie Universität Berlin, Humboldt-Universität Zu Berlin, and Berlin Institute of Health, Berlin, Germany; 2 NeuroCure Clinical Research Center, Charité—Universitätsmedizin Berlin, Corporate Member of Freie Universität Berlin, Humboldt-Universität Zu Berlin, and Berlin Institute of Health, Berlin, Germany; 3 Faculty of Psychology and Neuroscience, Maastricht University, Maastricht, Netherlands; 4 Einstein Center for Neurosciences, Berlin, Germany; 5 Department of Experimental Neurology and Center for Stroke Research, Charité—Universitätsmedizin Berlin, Berlin, Germany; 6 Department of Neurology, University of California, Irvine, CA USA

## Introduction

Fatigue is a poorly understood symptom in multiple sclerosis (MS), despite its high frequency and influence on the quality of life [1]. Studies suggest a multifactorial model, separating between primary MS-related and secondary factors [1, 2]. Primary factors entail disease activity and immune activation, in particular the influence of endocrines and proinflammatory cytokines [3–6], as well as alternations to brain areas suggestive to be involved in the perception of fatigue [7, 8]. Secondary mechanisms involve comorbidities such as additional autoimmune disease and psychiatric disorders [3, 4]. Multiple studies found poor sleep quality to be linked with higher fatigue levels and additionally associated with increased depression scores [9, 10]. Indeed, major depressive disorder (MDD) is one of the most common fatigue-influencing comorbidities in patients with MS [11, 12]. MDD is not only highly prevalent in MS patients but is also associated with immune system alterations and hyperactivity of the hypothalamic–pituitary–adrenal axis with subsequently altered inflammatory neuroendocrine factors and is thus directly linked to primary diseases mechanisms in MS [11, 13–16].

Our goal was to describe the evolution of fatigue in MS compared with a healthy population, especially in regard to primary and secondary factors associated with fatigue.

## Patients and methods

For this retrospective analysis, we selected patients and controls from ongoing observational cohort studies (Berlin-CIS-COHORT, NCT01371071, and VIMS, EA1/182/10). Inclusion criteria were a diagnosis of CIS or RRMS according to the 2010 McDonald criteria. Of 170 screened patients, 133 patients were included (CIS *N* = 100, RRMS *N* = 33, median follow-up time (time between first and last visit, inter-quartile range (IQR)): 2.40 years (1.45, 3.81)) and 30 healthy controls (HCs) matched for age (*p* = 0.784) and sex (*p* = 0.072). 16 HCs had no relevant comorbidities. Inclusion criteria were a baseline diagnosis of CIS or early RRMS according to the McDonald criteria (revised version 2010) [17] with less than 3 years since disease onset, and a minimum age of 18. Exclusion criterium was a missing FSS questionnaire. The cohort studies were approved by the local ethics committee and conducted in accordance with the Declaration of Helsinki. All participants gave written informed consent.

Disability was assessed using the expanded disability status scale (EDSS). Fatigue was assessed using the fatigue severity scale (FSS) and categorized into non-fatigued (FSS ≤ 4.0), borderline fatigued (4.0 < FSS < 5.0), or fatigued (FSS ≥ 5.0). Depressive symptoms and sleep quality were measured with Beck’s depression inventory (BDI-II, cut-off: 13) and the Pittsburgh Sleep Quality Index (PSQI, cut-off: 6), respectively.

Whole-brain lesion volume was obtained using the lesion segmentation toolbox with manual correction in ITK-SNAP using MPRAGE and FLAIR images of a 3 T MRI scanner (MAGNETOM Trio Tim Siemens, Erlangen, Germany).

Group comparisons were performed with *χ*^2^ (test statistic: *χ*^2^) or Wilcoxon test (test statistic: W). Correlations were analyzed using parametric or Spearman’s correlation. All tests were performed using R 3.6.0. *P* values < 0.05 were considered significant.

## Results

We found no differences in fatigue distribution between patients and HCs at baseline (HC vs. patients: *χ*^2^ = 3.135, *p* = 0.209) (Table 1).

**Table 1:** Cohort description

	HC	Patients
Subjects [*N*]	30	133
Subjects with longitudinal data [*N*]	16	133
Sex [m (%)]	15 (50.0)	44 (33.1)
Age at baseline [mean (SD)]	33.6 (12.0)	32.8 (9.1)
Follow-up time in years [median (IQR)]	2.0 [1.1, 2.7]	2.4 [1.5, 3.8]
Time since onset at baseline [months; median (IQR)]		4.0 [3.0, 5.0]
EDSS at baseline [median (IQR)]		1.5 [1.0, 2.0]
**BDI Score at baseline** [median (IQR) *N* = 80] (available for 30 HC and 50 patients)	0.50 [0.00, 6.75]	6.00 [2.00, 11.00]
Subjects without depressive symptoms at baseline [*N* (%)]	30 (100.0%)	46 (34.6%)
Subjects with depressive symptoms at baseline [*N* (%)]	0 (0.0%)	4 (3.0%)
**FSS Score at baseline** [median (IQR)]	2.34 [1.67, 2.92]	2.50 [1.53, 4.11]
Not fatigued subjects at baseline according to FSS [*N* (%)]	26 (86.7%)	99 (74.4%)
Borderline fatigued subjects at baseline according to FSS [*N* (%)]	3 (10.0%)	14 (10.5%)
Fatigued subjects at baseline according to FSS [*N* (%)]	1 (3.3%)	20 (15.0%)
**FSS score at last visit** [median (IQR)]	2.45 [1.67, 3.45]	2.28 [1.78, 3.81]
Not fatigued subjects at last visit [*N* (%)]	14 (87.5%)	99 (74.4%)
Borderline fatigued subjects at last visit [*N* (%)]	1 (6.3%)	12 (9.0%)
Fatigued subjects at last visit [*N* (%)]	1 (6.3%)	22 (16.6%)

*BDI* Beck’s Depression Inventory, *EDSS* expanded disability status scale, *FSS* Fatigue Severity Scale, *IQR* inter-quartile range, *m* male, *N* number, *SD* standard deviation

**Fig. 1:**
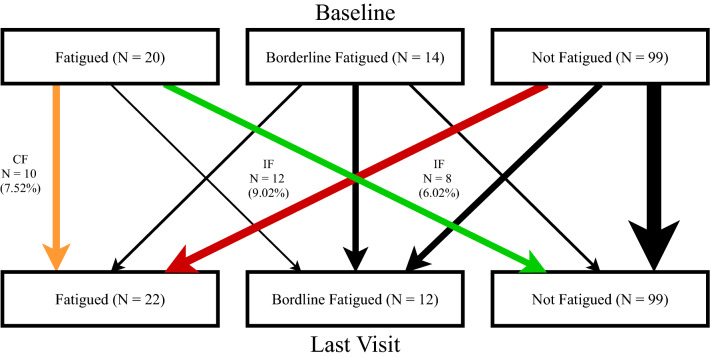
Tracking changes in individual patient fatigue status (*N* = 133) from baseline to follow up. Patients with a consistent fatigue categorization (CF) are highlighted in orange and patients changing from fatigued to non-fatigued or from non-fatigued to fatigued (IF) are highlighted in green and red, respectively. *CF* Patients with a consistent fatigue categorization, *IF* patients changing from fatigued to non-fatigued or from non-fatigued to fatigued, *N* number

During follow-up, 88 patients had a consistent fatigue status (fatigued = 10 [8%], non-fatigued = 78 [59%]). 45 (34%) patients changed fatigue status (inconsistent fatigue (IF)): Twelve patients lost their fatigue status at follow-up (9%), whereas 8 patients developed fatigue at follow-up (6%). The remaining patients showed only a minor change of scores and switched from or to borderline fatigued (Fig. 1, black arrows). The majority (13 [81.25%]) of HCs had a consistent fatigue status, with only one (6.25%) HC categorization changing from borderline to non-fatigued at follow-up. Fewer patients (*N* = 78; 59%) were consistently categorized as not fatigued than HCs (*N* = 13; 81%). More patients (*N* = 45; 34%) than HCs (*N* = 1; 6.25%) had an unstable fatigue status over time (*p* = 0.012, *χ*^2^ = 6.309).

We then analyzed primary and secondary factors in patients (*n* = 30) categorized as consistently high fatigued (CF, *n* = 10, Fig. 1, yellow arrow) or IF (*n* = 20, Fig. 1, green and red arrows). Regarding primary factors, 7 (23%) patients had an EDSS increase (≥ 1) at follow-up (4 CF, 3 IF). At baseline, EDSS was higher in fatigued than non-fatigued patients (*p* = 0.021). Changes in EDSS and in the fatigue group were associated (*p* = 0.042). In contrast, lesion volume did not correlate with fatigue at baseline (*R* = 0.680, *p* = 0.502) or follow-up (*R* = 0.847, *p* = 0.406) and was not associated with consistency of fatigue status ([W] 699.0, *p* = 0.774).

Investigating secondary mechanisms, the majority of IF (18 [90%]) and CF (9 [90%]) patients showed comorbidities. 8 IF (40%) and 6 CF (60%) had comorbid autoimmune disorders. Thyroid disorders were observed in 1 CF (10%) with Hashimoto-thyroiditis, 4 IF (20%) with hypothyroidism (2 IF (10%) with Hashimoto-thyroiditis, 1 IF (5%) after thyroidectomy due to focal thyroid autonomy, 1 IF (5%) not further classified) and 1 IF (5%) with an elevated Thyroid-Stimulating-Hormone level (not further classified). 9 IF (45%) and 8 CF (80%) showed psychiatric comorbidities: Depressive symptoms were present in 8 IF (40%) and 8 CF (80%). In patients, FSS and BDI-II were associated (*χ*^2^ = 27.6, *p* < 0.0001). Of the 22 patients (15 IF, 7 CF) with sleep quality data, 4 IF (27%) and 6 CF (86%) reported consistently poor sleep.

## Discussion

We observed a high prevalence and persistence of fatigue without a general alteration pattern in early MS compared with HCs. In our study at disease onset, only 15% of MS/CIS patients reported fatigue. This is in contrast to two previous studies from one center, which reported frequencies of up to 45% [18, 19]. Concerning primary fatigue, disability and disability worsening were associated with fatigue at onset and development of fatigue, whereas MRI T2-weighted lesion load did not contribute. Most relevant in our study were secondary factors of fatigue, namely concomitant autoimmune and mood disorders as well as poor sleep.

In contrast, primary mechanisms hardly are associated with a higher prevalence of fatigue [3, 14]. Thus, MS patients appear to be either prone to fatigue or stay stable not fatigued after their first event. Disease progression as the most important primary mechanism appears to play only a minor role since fatigue did not increase over time and was not associated with disease activity. However, increasing disability (e.g. newly developed sensory disorder or spasticity) might lead to perceived mobility difficulties, which could be felt as weakness or be interpreted as fatigue. As many patients are stable, not fatigued even though EDSS increases, a direct link between disability and fatigue is unlikely. Studies supporting this multifactorial highly interactive disease model have further found psychiatric comorbidities, such as mood and anxiety disorders, to be associated with higher EDSS scores leading to the assumption that also these comorbidities confound the correlation of EDSS and fatigue [12, 20–22].

Limitations of this study include the lack of data prior to the MS diagnosis and the shorter follow-up in HC. The low prevalence of fatigue may limit our sample’s representativeness.

To conclude, secondary factors have to be taken into account when assessing and managing fatigue in MS. These comorbidities may open an important avenue of interventions in patients suffering from fatigue and should thus be evaluated before considering treatment options for primary fatigue. In contrast, primary factors of fatigue were less important in our sample of patients at disease onset.

## Data Availability

The data that support the findings of this study are available on reasonable request.
